# Delayed-Onset Hemolytic Anemia in Patients with Travel-Associated Severe Malaria Treated with Artesunate, France, 2011–2013

**DOI:** 10.3201/eid2105.141171

**Published:** 2015-05

**Authors:** Stéphane Jauréguiberry, Marc Thellier, Papa Alioune Ndour, Flavie Ader, Camille Roussel, Romain Sonneville, Julien Mayaux, Sophie Matheron, Adela Angoulvant, Benjamin Wyplosz, Christophe Rapp, Thierry Pistone, Bénédicte Lebrun-Vignes, Eric Kendjo, Martin Danis, Sandrine Houzé, François Bricaire, Dominique Mazier, Pierre Buffet, Eric Caumes, French Artesunate Working Group

**Affiliations:** Université Pierre et Marie Curie, Paris, France (S. Jauréguiberry, M. Thellier, P.A. Ndour, C. Roussel, M. Danis, D. Mazier, P. Buffet, E. Caumes);; Hôpital Pitié-Salpêtrière, Assistance Publique des Hôpitaux de Paris (APHP), Paris (S. Jauréguiberry, M. Thellier, F. Ader, J. Mayaux, B. Lebrun-Vignes, M. Danis, F. Bricaire, D. Mazier, P. Buffet, E. Caumes);; Centre National de Référence du Paludisme, Paris (S. Jauréguiberry, M. Thellier, E. Kendjo, M. Danis, D. Mazier, P. Buffet);; Hôpital Bichat, APHP, Paris (R. Sonneville, S. Matheron, S. Houzé);; Université Paris-Sud, Orsay, France (A. Angoulvant);; Hôpital de Bicêtre, Le Kremlin Bicêtre, APHP, France (A. Angoulvant, B. Wyplosz);; Hôpital d’Instruction des Armées Begin, St. Mandé, France (C. Rapp);; Hôpital Pellegrin, Bordeaux, France (T. Pistone)

**Keywords:** hemolysis, hemolytic anemia, malaria, artesunate, delayed-onset, safety, therapeutic benefit, incidence, outcome, travelers, imported, travel-associated, vector-borne infections, parasites, Plasmodium falciparum, France

## Abstract

Artesunate is the most effective treatment for severe malaria. However, delayed-onset hemolytic anemia has been observed in ≈20% of travelers who receive artesunate, ≈60% of whom require transfusion. This finding could discourage physicians from using artesunate. We prospectively evaluated a cohort of 123 patients in France who had severe imported malaria that was treated with artesunate; our evaluation focused on outcome, adverse events, and postartesunate delayed-onset hemolysis (PADH). Of the 123 patients, 6 (5%) died. Overall, 97 adverse events occurred. Among the 78 patients who received follow-up for >8 days after treatment initiation, 76 (97%) had anemia, and 21 (27%) of the 78 cases were recorded as PADH. The median drop in hemoglobin levels was 1.3 g/dL; 15% of patients with PADH had hemoglobin levels of <7 g/dL, and 1 required transfusion. Despite the high incidence of PADH, the resulting anemia remained mild in 85% of cases. This reassuring result confirms the safety and therapeutic benefit of artesunate.

## Abstract 2

Hemolysis occurred in a low proportion of patients and did not increase transfusion requirements.

Intravenous (IV) artesunate has been the recommended first-line treatment for severe malaria worldwide since 2010 (*1*). Two large randomized trials showed a 35.0% reduction (from 22.0% to 15.0%) in death rates among adults in Asia and a 22.5% (from 10.9% to 8.5%) reduction among children in Africa when artesunate was compared with parenteral quinine in the treatment of severe malaria (*2*,*3*). Four case series performed in Western countries reported death rates of <4% (*4*–*7*).

Artesunate is generally considered safe (*8*). However, its use in Western countries has shown that delayed hemolytic events occur in ≈20% of patients with severe imported malaria, and 60% of these patients require blood transfusion (*4*,*6*,*7*,*9*–*11*). Delayed-onset anemia (herein referred to as postartesunate delayed-onset hemolysis [PADH] pattern of anemia) has been observed to occur 2–3 weeks after initiation of IV artesunate, after complete clearance of parasites, and to resolve during weeks 3–6 (*7*). The mechanism of this anemia is hemolytic, as demonstrated by high serum lactate dehydrogenase (LDH) and low plasma haptoglobin levels. Across several studies, no common conventional cause of hemolysis was identified (*4*,*6*,*12*–*14*). In a comparative study, PADH anemia was described in 5 of 8 patients with hyperparasitemia treated with artesunate alone or combined with quinine; it was not seen in patients treated with quinine alone. This finding supports the assumption that this side effect is associated with artesunate (*11*). PADH anemia has not been reported in meta-analyses (*8*) nor observed in large clinical trials (*2*,*3*). However PADH has been reported recently in children in Africa (*15*).

This PADH is a matter of concern for the medical community. Without a systematic assessment of the incidence and outcome of artesunate-associated PADH anemia, a slowdown may occur in the ongoing change toward favoring treatment with artesunate rather than quinine, a less-efficient treatment for severe malaria. The World Health Organization recently recommended increased vigilance for PADH anemia and called for a more precise description of its incidence, time course, and severity (*16*). To determine the effectiveness and safety of artesunate in patients with severe imported malaria, we focused on PADH anemia cases detected through an existing artesunate surveillance program in France.

## Materials and Methods

### Temporary Use Authorization Program and Treatment

In May 2011, IV artesunate (60-mg vial of powder and solvent) became available in France through the Agence Nationale de Sécurité du Médicament, the French national drug agency. The product, manufactured by Guilin Laboratories in China, was imported to Europe by ACE Pharmaceuticals. Within the framework of a temporary use authorization program, data were prospectively collected during May 2011–May 2013 from medical charts and by using Agence Nationale de Sécurité du Médicament forms that were completed by attending physicians at the beginning and end of treatment. A dedicated team at the National Reference Center for Malaria (NRCM) retrieved the data. Additional data were obtained from the national pharmacovigilance system and an NRCM database, as described (*17*). Retrieved data included age, sex, native country, place of malaria acquisition, immunocompromised status, pregnancy status, appropriateness of chemoprophylaxis, purpose of travel, duration of disease before treatment, location where artesunate was prescribed, drug used as first-line treatment, median duration/dose of artesunate treatment, duration of hospitalization, outcome, clinical and biologic criteria for severe malaria, and duration of follow-up. The artesunate database was implemented and informed consent was obtained from patients in accordance with a procedure common to all French National Reference Centers (http://www.legifrance.gouv.fr/affichTexte.do?cidTexte=JORFTEXT000000810056&dateTexte=&categorieLien=id). Data were collected and analyzed anonymously. After diagnosis, all patients received IV artesunate (2.4 mg/kg) at 0, 12, and 24 hours and daily thereafter until oral antimalarial treatment could be administered to complete treatment as recommended in France (*18*). For patients who received a full 7-day course of IV artesunate, no other treatment was administered.

### Case Definitions

Severe malaria was defined as malaria in persons with blood smears positive for asexual forms of *Plasmodium falciparum* parasites and at least 1 criterion of severity according to the definition of severe malaria used in France (*19*,*20*). Patients with mixed-species infections were excluded from analysis.

Anemia was defined as a blood hemoglobin level of <12 g/dL in female and <13 g/dL in male patients (reference values 12.0–16.5 and 13.0–17.5 g/dL, respectively). Hemolysis was defined as a plasma haptoglobin level of <0.1 g/L (reference value 0.55–2.50 g/L), plasma lactic dehydrogenase (LDH) level of >390 IU/L (reference value 190–390 IU/L), or both. We defined 3 patterns of anemia as previously described (*7*,*10*,*11*): PADH, non-PADH, and indeterminate. The PADH pattern was defined by 1) a new drop in the hemoglobin level after day 8 of treatment initiation and the appearance or reappearance of hemolytic markers (>10% drop in hemoglobin or >10% rise in LDH levels) occurring any time between day 8 and the end of follow-up and/or 2) by any information in the medical chart referring to acute hemolysis occurring after day 8. The non-PADH pattern was defined by a hemoglobin nadir and a hemolysis peak occurring before day 8, with or without positive markers of hemolysis after day 8 and without a nadir or sudden drop of hemoglobin after day 8 as defined for the PADH pattern. The indeterminate pattern was defined as all other cases of anemia for which information was lacking or with an evolution pattern that did not fit the other patterns.

### Sample Collection

Blood samples were routinely collected from the patients on days 0, 2 (±1), 7 (±2), 14 (±3), 21 (±3), and 28 (±3) after treatment initiation (*18*,*21*). Samples were analyzed to determine the levels of hemoglobin, total bilirubin, glucose, plasma bicarbonate, lactate, serum creatinine, blood urea nitrogen, LDH, haptoglobin, and parasitemia and the reticulocyte count. 

### Cure, Evolution, and Side Effects

The death rate at day 28 was the main clinical endpoint. Parasitological cure was defined as a *P*. *falciparum*–negative blood smear on day 7, with possible confirmation later. Relapse was defined as the reappearance of fever and a blood smear positive for asexual *P. falciparum* parasite forms at any time after a first negative result during the 28-day follow-up period. All side effects reported on medical charts were recorded and graded by using the National Institutes of Health grading system (http://www.niaid.nih.gov/LabsAndResources/resources/DAIDSClinRsrch/Documents/daidsaegradingtable.pdf).

### Statistical Analyses

Travel characteristics and demographic, clinical, and laboratory variables were evaluated. Quantitative variables were expressed as medians (quartiles 1–3 [Q1–3]) or, when appropriate, as means (SEMs). Qualitative variables were expressed as percentages. Differences between groups (survivors vs. nonsurvivors, patterns of anemia) were analyzed by using the Fisher exact test for categorical variables and Mann-Whitney test for continuous variables. Statistical analyses were performed by using IBM SPSS Statistics version 20 (IBM, Armonk, NY, USA). All reported p values are 2-tailed.

## Results

### General Presentation of Cohort

**Figure 1:**
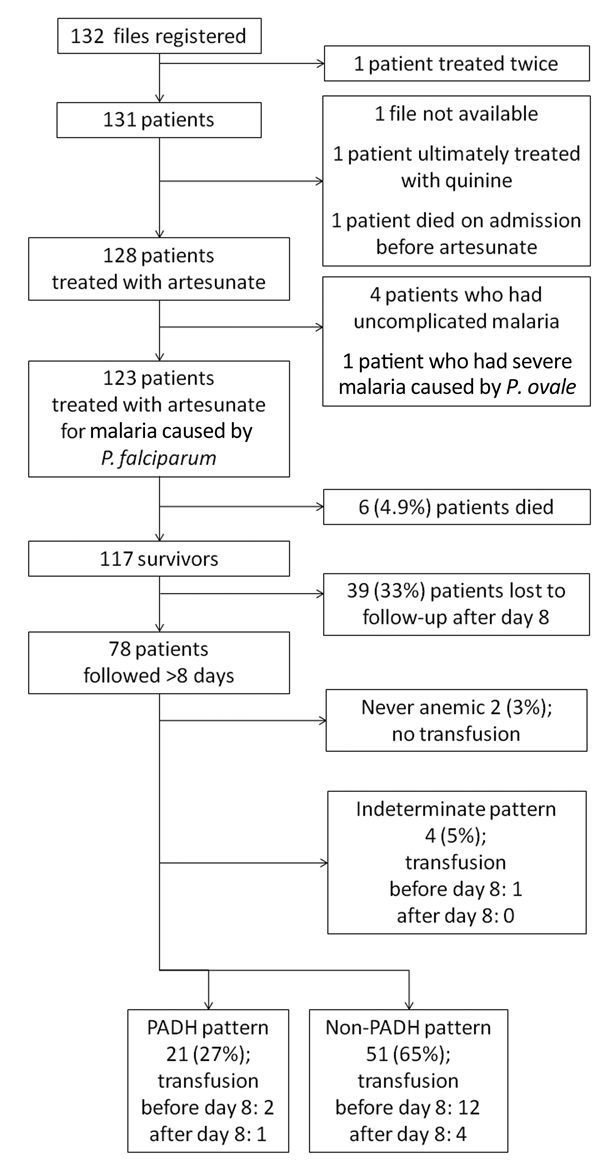
Distribution of PADH and non-PADH patterns of anemia in a prospective analysis of delayed-onset hemolytic anemia in patients with severe imported malaria treated with artesunate, France, 2011–2013. Of 123 patients who received treatment, 6 died and 39 were lost to follow-up after day 8, leaving a total of 78 patients with sufficient clinical and/or biologic information to fulfill the anemia definition criteria for PADH or non-PADH classification. Indeterminate pattern, cases of anemia for which information was lacking or with an evolution pattern that did not fit PADH or non-PADH patterns; PADH, postartesunate delayed-onset hemolysis.

A study flowchart is provided in Figure 1. Demographic and clinical characteristics of the 123 patients who received artesunate treatment are summarized in Technical Appendix Table.

### Effectiveness

Among the 123 patients with severe malaria treated with artesunate, 117 fully or partially recovered and 6 died from malaria (death rate 4.9%, 95% CI 2.0–10.8). All deaths were related to severe multiorgan failure and occurred within 3 days of receiving artesunate. 

The following characteristics were seen more frequently at admission in patients who died versus those who survived: lower median Glasgow Coma Scale (median score 10 [Q1–3: 3–13]) vs. 14 [Q1–3: 14–15], p = 0.001; reference score 15); respiratory distress (50% [3/6] vs. 9% [11/117], p = 0.019); higher median parasitemia level (11% [Q1–3: 8–26] vs. 6% [Q1–3: 2–10], p = 0.05); higher median lactate level (10 mmol/L [Q1–3: 3–12] vs. 2 mmol/L [Q1–3: 2–3], p = 0.002; reference value <1.8 mmol/L); higher total bilirubin concentration (98 μmol/L [Q1–3: 98–209] vs. 49 μmol/L [Q1–3: 26–75], p = 0.007; reference value 2–17 mmol/L); lower glucose level (<2.2 mmol/L in 50% [3/6] vs. <2.2 mmol/L in 3% [4/117], p = 0.002; reference value 3.9–5.8 mmol/L); and renal insufficiency (80% [5/6] vs. 9% [11/117], p<0.001). Age, sex, immunocompromised state, place of malaria acquisition, cardiocirculatory impairment (p *=* 0.098), and severe anemia at day 0 were not significantly associated with death. The median time between symptom onset and initiation of artesunate treatment was 1.5 days (Q1–3: 1–5) in the 6 patients who died versus 4.0 days (Q1–3: 2–5) in those who survived (p = 0.13). Artesunate was used as second-line treatment after quinine in 2 of 6 patients who died versus 49 of 117 patients who survived (p = 1). All survivors had complete parasite clearance before treatment day 7. Only 1 relapse was observed; it occurred 26 days after a 3-day course of IV artesunate that was not followed by the recommended oral course of antimalarial drug therapy.

### Safety

Safety data were available for days 0–8 and 9–28 for 123 and 78 patients, respectively. All reported adverse events resolved during follow-up. A summary of the severity of reported adverse events possibly associated with artesunate is shown in Table 1. 

**Table 1:** Severity of reported adverse events possibly associated with artesunate treatment of severe imported malaria in 117 patients, France, 2011–2013*

Adverse event	Grade 1, mildly severe	Grade 2, moderately severe	Grade 3, severe	Grade 4, life threatening	Total
Cutaneous	2†	1‡	0	0	3
Ataxia, tremor, CNS ischemia	0	2	1	1	4
Tinnitus	1	0	0	0	1
Cardiac and arterial ischemia	1	1	1	1	4
Hypertension	0	0	1§	0	1
Elevated level of ALT	2	2	3	1	8
Hyperkalemia	1	0	0	0	1
Anemia¶	20	11	27	17	75
Total	27	17	33	20	97


*Adverse events were obtained from medical charts and graded by using the National Institutes of Health classification for side effects (http://www.niaid.nih.gov/LabsAndResources/resources/DAIDSClinRsrch/Documents/daidsaegradingtable.pdf). ALT, alanine transaminase; CNS, central nervous system. †Mild pruritus and rash. ‡Telogen effluvium. §Regressive retinopathy. ¶Grading was done during the follow-up by using available nadir of hemoglobin. For HIV-negative patients, the severity of anemia was graded as follows: grade 1, 10.0–10.9 g/dL; grade 2, 9.0–9.9 g/dL; grade 3, 7.0–8.9 g/dL, grade 4, <7.0 g/dL. For HIV-positive patients, the severity of anemia was graded as follows: grade 1, 8.5–10.0 g/dL; grade 2.0, 7.5–8.4 g/dL; grade 3, 6.5–7.4 g/dL; grade 4, <6.5 g/dL.

Rash, telogen effluvium, and mild pruritus were recorded for 1 patient each. The rash occurred several days after the end of artesunate administration and was considered unrelated to artesunate. Telogen effluvium was diagnosed during the 28 days following treatment initiation. The pruritus occurred during artesunate treatment and disappeared without intervention. 

Liver enzyme levels increased in 8/117 patients who survived, including 6 who concurrently received >1 medication(s) with liver toxicity as a potential side effect (paracetamol [acetaminophen] or nonsteroidal antiinflammatory drug). All these episodes occurred before day 8. Vision loss occurred in 1 patient and was considered by the attending ophthalmologist to be associated with hypertensive retinopathy. Two cases of acute cerebellar syndrome occurred; both were thought to be associated with a postmalaria neurologic syndrome. Tinnitus was reported in 1 patient who received quinine just before artesunate. One patient experienced continuous tremors that resolved spontaneously. 

QTc lengthening (i.e., corrected lengthening of the interval between start of the Q wave and end of the T wave in the heart’s electrical cycle) and transient bradycardia were recorded for 1 and 2 patients, respectively. One transient bradycardia (54 bpm) episode occurred between 2 artesunate injections and resolved spontaneously. In the patient with QTc lengthening (460 ms; reference value <440 ms), artesunate treatment (total dose 480 mg) was changed to artemether/lumefantrine (4 tablets, each with 20 mg artemether and 120 mg lumefantrine) and then to atovaquone/proguanil (4 tablets/d for 3 d, each with 250 mg atovaquone and 100 mg proguanil) because of persistent QTc lengthening (560 ms) accompanied by low potassium levels (<3 mmol/L; reference range 3.5–5.0 mmol/L). These 2 patients were 13 and 15 years of age and weighed 62 kg and 40 kg, respectively. Another patient experienced severe disseminated intravascular coagulation that led to arterial ischemia of extremities and central nervous system ischemia (caudate nuclei, corona radiate, and white matter). Amputation of fingers and legs was necessary. All sequelae in this patient were considered related to severe malaria. 

One patient with myasthenia gravis received artesunate and experienced no worsening of the disease (*22*). Four patients were pregnant; 1 of the pregnancies was discovered during therapy. One miscarriage occurred; hemorrhage led to a blood transfusion. Artesunate was well tolerated in 2 women during the second and third trimesters and in 1 during labor. Hypoglycemia was not recorded during artesunate treatment.

### Anemia, Hemolysis, and Transfusion

**Figure 2:**
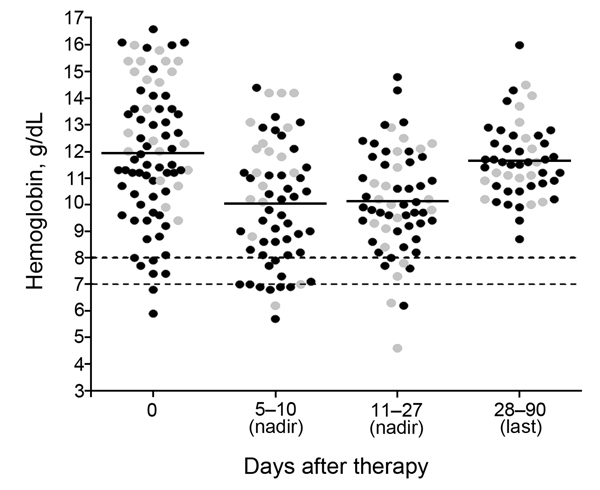
Nadir and last hemoglobin levels for 78 patients in a prospective analysis of delayed-onset hemolytic anemia in patients with severe imported malaria treated with artesunate, France, 2011–2013. Gray dots, hemoglobin level for patients with the postartesunate delayed-onset hemolysis (PADH) pattern of anemia; black dots, hemoglobin level for patients with non-PADH pattern of anemia, indeterminate pattern and nonanemic patients. Dotted lines represent hemoglobin level thresholds of 8 or 7 g/dL.

Anemia commonly occurred, was slow to resolve, and followed variable patterns (Figures 1, 2). Of the 78 patients with appropriate follow-up, 76 (97.4%, 95% CI 91.0%–99.7%) experienced anemia (Figure 1). The non-PADH and PADH patterns were observed in 51 (65.4%, 95% CI 53.8%–75.8%) and 21 (26.9%, 95% CI 17.5%–38.2%) patients, respectively, with or without transfusion, who received clinical and/or laboratory follow-up beyond day 8. In the PADH group, hemoglobin levels dropped a median of 1.3 g/dL from day 7 (±2) to day 14 (±3), but levels ranged from 4.6 g/dL to 12.9 g/dL on day 14 (±3). During days 11–27, a total of 8 patients had severe anemia, of whom 4 experienced typical PADH anemia (p = 0.4) (Figure 2). Of the 21 total patients with PADH, 3 (14.3%, 95% CI 3.1%–36.3%) had blood hemoglobin levels of <7 g/dL (6.2, 4.6, and 6.3 g/dL) during week 2 after treatment initiation.

**Figure 3:**
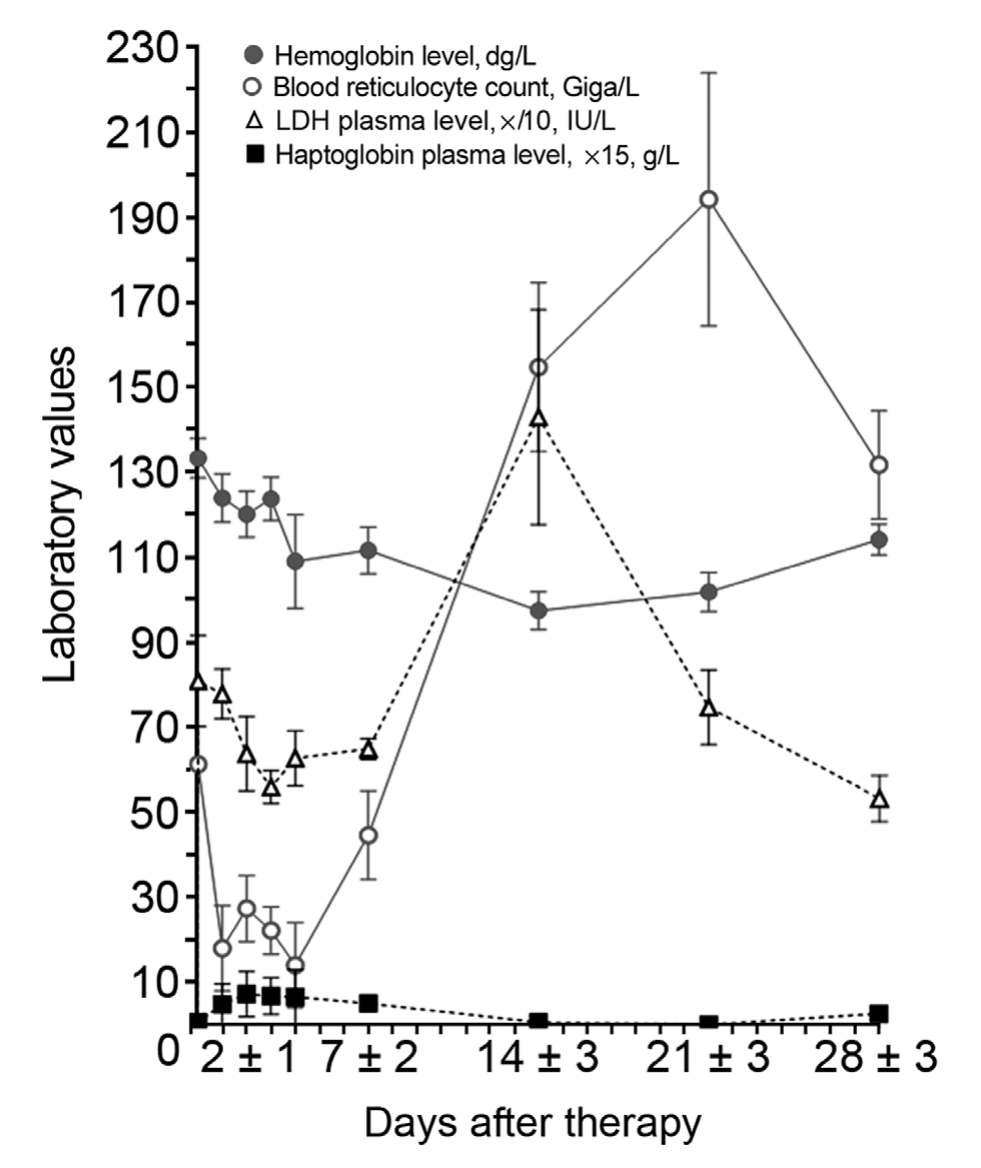
Typical features of postartesunate delayed-onset hemolysis and anemia for 21 patients followed in a prospective analysis of delayed-onset hemolytic anemia in patients with severe imported malaria treated with artesunate, France, 2011–2013. During the second and third weeks of late hemolytic anemia, a drop in hemoglobin occurred along with a reoccurrence of markers of hemolysis (defined as >10% drop in hemoglobin level or >10% rise in LDH concentration). Reticulocyte regeneration occurred during week 3 (delayed in comparison with other patterns of anemia, in which regeneration usually occurs during week 2 [not shown]). Values are means (SEMs). LDH, lactate dehydrogenase.

With the exception of hemoglobin level, no parameters were significantly associated with a particular anemia pattern (Table 2; Technical Appendix Table 2); median hemoglobin levels at day 0 were estimated to be 11.3 g/dL (Q1–Q3: 9.6–13.1) and 13.6 g/dL (Q1–Q3: 11.6–15.4), respectively, for patients with non-PADH and PADH anemia (p *=* 0.002) (Tables 2, 3). The incidence of the PADH anemia did not differ between patients who received artesunate as first-line treatment and those who received quinine before being switched to artesunate (p = 0.38, Fisher exact test). During week 2, the rise in LDH levels and the drop in haptoglobin levels were consistent with hemolytic anemia in the PADH group (Figure 3); these changes lasted ≈1–2 weeks. Median LDH levels remained high at days 21 (724 IU/L [range 344–1,564] and 28 (497 IU/L [range 177–922]). Haptoglobin remained undetectable during weeks 2 and 3 (Table 3; Figure 3). During subsequent weeks, hemoglobin levels rose slowly in the non-PADH group (Table 3). Maximum reticulocyte production occurred during weeks 2 and 3 for the non-PADH and PADH groups, respectively (Figure 3; Table 3). At day 28, patients in both groups had hemoglobin levels >11 g/dL.

**Table 2:** Association between selected variables and non-PADH and PADH patterns of anemia in 72 patients with severe imported malaria treated with artesunate, France, 2011–2013*

Variable	Pattern		p value
Non-PADH, n = 51†	PADH, n = 21‡
Sex, no. (%)			
M	29 (57)	11 (52)	0.8§
F	22 (43)	10 (48)	
Age, y, median (Q1–Q3)	42 (27–52)	41 (31–53)	0.7¶
Location of birth, no. (%)			
Africa	31 (61)	10 (48)	0.3§
Europe, North America	18 (35)	11 (52)	
South, Central America	2 (4)	0	
Asia	0	0	
Duration of illness before artesunate treatment, median d (Q1–Q3)	4 (3–5)	4 (3–5)	0.8¶
Hyperparasitemia, no. (%)			
>4% infected erythrocytes	28 (55)	16 (76)	0.1§
>10% infected erythrocytes	13 (25)	5 (24)	1§
Parasitemia level at day 0, median % infected erythrocytes (Q1–Q3)	5.0 (1.4–10.1)	7.1 (3.75–14.5)	0.1¶
Hemoglobin level, median g/dL (Q1–Q3), at day 0	11.3 (9.6–13.1)	13.6 (11.6–15.4)	0.002¶
Total dose of artesunate, median mg (Q1–Q3)	840 (540–1035)	800 (676–955)	0.8¶
Artesunate first-line treatment for current severe malaria episode, no. (%)	29 (57)	15 (71)	0.3§

*PADH, postartesunate delayed-onset hemolysis; Q1–Q3, quartiles 1–3. †Non-PADH pattern was defined by a hemoglobin nadir and a hemolysis peak occurring before day 8, with or without positive markers of hemolysis after day 8 and without a nadir or sudden drop of hemoglobin after day 8. ‡PADH pattern was defined by 1) a new drop in the hemoglobin level after day 8 of treatment initiation and the appearance or reappearance of hemolytic markers occurring any time between day 8 and the end of follow-up and/or 2) by any information in the medical chart referring to acute hemolysis occurring after day 8. §Fisher exact test. ¶Mann-Whitney test.

**Table 3:** Laboratory values for 72 patients with artesunate-treated severe imported malaria and a PADH or non-PADH pattern of anemia during days 0–28 after treatment initiation, France, 2011–2013*

Patient group, laboratory test	Median value (range), no. results available				
	Day 0	Day 7	Day 14	Day 21	Day 28
Non-PADH†					
Hemoglobin level, g/dL‡	11.3 (5.9–16.6), 51	9.2 (5.7–13.1), 62	9.9 (6.2–14.3), 32	10.6 (7.7–13.0), 22	11.5 (8.2–13.9), 30
Reticulocyte count, G/L	61 (3–183), 16	60 (2–444), 25	156 (75–412), 20	108 (56–204), 12	73 (34–100), 12
LDH level, IU/L	777 (161–3,003), 24	803 (312–2,722), 28	537 (261–1,139), 17	521 (201–905), 12	464 (240–798), 15
Haptoglobin level, g/L	0.05 (0.00–2.20), 8	0 (0.00–2.90), 25	0 (0.00–2.50), 20	0 (0.00–2.00), 10	0.40 (0.00–1.40), 12
PADH§					
Hemoglobin level, g/dL¶	13.6 (9.4–16.0), 21	11.2 (6.2–14.2), 20	9.9 (4.6–12.9), 24	10.0 (6.7–13.4), 15	11.1 (10–15), 17
Reticulocyte count, G/L	61 (61–61), 1	34 (8–132), 12	124 (63–315), 15	162 (90–431), 12	127 (63–223), 13
LDH level, IU/L	846 (293–1,195), 9	634 (510–793), 14	1,128 (554–4,000), 17	724 (344–1,564), 13	497 (177–922), 14
Haptoglobin level, g/L	0 (0.00–0.20), 5	0 (0.00–1.50), 15	0 (0.00–0.60), 18	0 (0.00–0.00), 13	0 (0.00–1.50), 15

*G/L, Giga/L; LDH, lactate dehydrogenase; PADH, postartesunate delayed-onset hemolysis.  †Non-PADH pattern was defined by a hemoglobin nadir and a hemolysis peak occurring before day 8, with or without positive markers of hemolysis after day 8 and without a nadir or sudden drop of hemoglobin after day 8.  ‡Median drop in hemoglobin during the first week of treatment was 2.1 g/dL. §PADH pattern was defined by 1) a new drop in the hemoglobin level after day 8 of treatment initiation and the appearance or reappearance of hemolytic markers (defined as >10% drop in hemoglobin or >10% rise in LDH levels) occurring any time between day 8 and the end of follow-up and/or 2) by any information in the medical chart referring to acute hemolysis occurring after day 8. ¶Median drop in hemoglobin during the first week of treatment was 2.4 g/dL.

Of the 78 patients, 15 (19.3%, 95% CI 10.5%–28.0%) received a total of 20 blood transfusions, 15 (75%) of which were performed before day 8, during the acute phase of the disease (Figure 1). Among the 21 patients with delayed hemolysis, 1 (4.8%, 95% CI 0.1%–23.8%) had a blood transfusion after day 8 (hemoglobin nadir 6.3 g/dL). No deaths were related to any side effects, including anemia.

## Discussion

According to NRCM data, each year in France, ≈250 patients are treated for severe imported malaria. In this cohort of 123 patients treated for severe malaria in high-care settings, IV artesunate was effective and generally safe. The death rate was 5%, and blood transfusion was necessary for <20% of all patients and for <5% of patients with PADH anemia. Compared with retrospective case series, our prospective approach reduced bias toward severe anemia cases and provided a robust evaluation of artesunate safety, particularly as concerns PADH anemia.

The 5% death rate in this cohort is lower than rates observed among artesunate-treated adults who received treatment in malaria-endemic countries (*2*,*3*) but similar to rates among smaller cohorts of travelers from non–malaria-endemic countries whose treatment was managed in facilities with high levels of care (*23*,*24*). A retrospective study in the United Kingdom that compared 143 quinine-treated patients with 24 artesunate-treated patients reported no deaths, fewer intensive care unit admissions, and shorter durations of hospitalization for artesunate-treated patients (*25*). In a study of 400 severe imported *P. falciparum* malaria cases treated with quinine (10% death rate), the major factors associated with death were low Glasgow Coma Scale score, respiratory failure, severe renal impairment, hyperlactemia, or hypoglycemia (*23*) during the first 24 hours after admission. Despite our use of different data-capture methods and severity scores, we found the same factors associated with death in this cohort of artesunate-treated patients.

PADH occurred in 27% of patients in this study, but it was rarely associated with severe anemia and was never fatal. Previous observations (*7*) may have been partially biased toward the most severe cases; in contrast, our prospective approach efficiently captured asymptomatic mild or moderate anemia cases. In our study, the median delayed drop in hemoglobin levels was 1.3 g/dL. Although PADH accounted for most of the severe anemia cases (Figure 2), only 3 (15%) patients had hemoglobin levels of <7 g/dL, and only 1 received a transfusion. This transfusion rate (<5%) is markedly lower than that previously reported for patients with severe imported malaria and delayed-onset anemia (≈60%) (*4*,*6*,*7*,*11*,*13*). Taken together, our results demonstrate that, in the setting of severe imported malaria, delayed hemolysis does not alter the life-saving effect of IV artesunate, but it does need focused medical attention and follow-up.

With the exception of the pretreatment hemoglobin level, no parameters, including the cumulative dose of artesunate and initial parasitemia levels, were correlated with the risk for delayed hemolysis in our study. Some authors have associated high parasitemia levels with delayed-onset hemolysis (*6*,*7*,*11*); others have demonstrated that not all patients with high parasitemia levels experience late-onset hemolysis (*4*). Other factors are related to the peculiar mode of action linked to artemisinin derivatives and are probably involved in delayed-onset hemolysis (*4*,*26*). Indications pointing to the involvement of pitting (*27*,*28*), a process whereby dead parasites are expelled from infected erythrocytes, has been reported (*29*,*30*). We have shown that the risk for PADH is linked to the peak number of pitted erythrocytes rather than the absolute initial level of parasitemia (*31*).

Side effects of artesunate frequently include gastrointestinal disturbances, neutropenia (1.3%), reticulocytopenia (0.6%), and elevated liver enzymes (1.1%) (*32*–*34*). In studies of patients with travel-associated malaria treated in non–malaria-endemic countries, no severe hemodynamic, cardiac, or allergic reactions were attributed to artesunate (*4*,*5*,*7*,*11*). Artesunate is considered potentially cardiotoxic at doses >15 mg/kg (*32*). However, in our study, mild to moderate cardiotoxicity developed in 3 patients treated with the recommended 2.4-mg/kg dose. The patients were rapidly switched to another therapy, and the signs and symptoms of cardiotoxicity disappeared. Whether these cardiac episodes were related to artesunate is not clear. In a study performed in Bangladesh involving 21 adults with severe malaria treated with artesunate, 2 patients experienced QTc lengthening (>500 ms), but, as observed in 1 of our patients, hypokalemia was present (*35*). None of the other side effects reported as possibly attributable to artesunate in other studies (e.g., dizziness, nausea, diarrhea, anorexia, metallic taste in the mouth) were recorded in this cohort, and the neurologic episodes were considered to be related to malaria rather than artesunate by the attending physicians. As reported for artesunate-associated side effects in other studies, those in our study were generally mild.

This study has limitations. The prospective surveillance system implemented in France relies on the motivation of attending physicians and parasitologists to report data to the NRCM team because the reporting of imported malaria cases is not compulsory in metropolitan France. Thus, some adverse events might have been overlooked. In addition, patients with PADH whose anemia was well tolerated may have gone unreported or been lost to follow-up. Furthermore, symptoms of slight delayed anemia may be confused with slow clinical recovery from severe malaria and thus remain undetected. In addition, it is likely that the 39 patients who were lost to follow-up recovered without problems instead of remaining as postinfectious patients with continuing problems. A rapid analysis, excluding patients who died, did not show any differences in demographic variables or length of hospital/intensive care unit stay between the group that was followed for 28 days and the group that was followed <8 days (data not shown). Nevertheless, it is unlikely that severe cases of delayed hemolysis or other severe side effects would be overlooked in a temporary use authorization program implemented at facilities with high levels of care, and, as mentioned, this study captured a fairly high proportion of mild to moderate cases of delayed hemolysis. Furthermore, the study was not randomized, but in the setting of severe imported malaria, it is considered unethical to repeat artesunate versus quinine trials already performed in malaria-endemic countries (*3*,*36*). The decision to use artesunate or quinine was left to the attending physician, but use of artesunate was mostly related to its availability at the hospital. It is unlikely that the decision to use/not use artesunate as a first-line treatment was made according to the clinical severity of disease in a patient.

Our prospective analysis joins other reports (*16*,*37*) in confirming the very favorable risk-to-benefit ratio of IV artesunate in the treatment of severe imported malaria, despite PADH anemia. Our results show that, in this setting, delayed hemolysis did not alter the life-saving effect of IV artesunate. Delayed hemolysis was a common occurrence among the patients but resulted in very low levels of hemoglobin in ≈15% of cases. To ensure the appropriate diagnosis and treatment of severe anemia, the World Health Organization and national entities recommend that hemoglobin levels be assessed weekly for 1 month after artesunate administration (*16*,*18*,*21*). Further studies are needed to find predictive markers of hemolysis and anemia to facilitate posttreatment follow-up in travelers to and children in malaria-endemic countries (*10*–*12*,*31*).

Technical Appendix Baseline data and complementary qualitative features of anemia for patients with severe imported malaria treated with artesunate, France, 2011–2013.
