# Supplementary material for: Delayed-Onset Hemolytic Anemia in Patients with Travel-Associated Severe Malaria Treated with Artesunate, France, 2011–2013
Source: Emerg Infect Dis. 2015 May;21(5):804–12. doi: 10.3201/eid2105.141171 (PMC4412216; doi:10.3201/eid2105.141171)
Supplement: Technical Appendix — Baseline data and complementary qualitative features of anemia for patients with severe imported malaria treated with artesunate, France, 2011–2013. [file 14-1171-Techapp-s1.pdf]

Article DOI: http://dx.doi.org/10.3201/eid2105.141171

Delayed-Onset Hemolytic Anemia in Patients with Travel-Associated Severe Malaria Treated with Artesunate, France, 2011–2013

Technical Appendix

Technical Appendix Table 1. Baseline (Day 0) demographics, travel characteristics, clinical and biologic data of 123 patients with imported severe malaria treated by artesunate, France, 2011–2013*

Characteristic; value for last column	Total no. patients	No. (%) patients	Median (Q1–Q3)
Sex	123		
M		74 (60)	
F		49 (40)	
Age, y; median age	123		42 (30–55)
0–15		9 (7)	
16–30		22 (19)	
31–49		48 (39)	
>50		45 (36)	
Area of birth	123		
Africa		65 (53)	
Europe, North America		54 (44)	
South, Central America–Caribbean		3 (2)	
Asia		1 (1)	
Place malaria acquired	123		
West Africa		116 (94)	
East and Austral Africa		6 (5)	
South America		1 (1)	
Immunocompromised status	123		
HIV		9 (7)	
Cirrhosis		1 (1)	
None		113 (92)	
Pregnancy	49	4 (8)	
Chemoprophylaxis during travel	123		
Yes		24 (20)	
Chloroquine		3	
Chloroquine + proguanil		2	
Mefloquine		2	
Atovaquone + proguanil		7	
Doxycycline		7	
Unknown prophylaxis		3	
Inadequate for country of travel		5	
Reason for stay in malaria-endemic zone; median length of stay, d	123		
Visiting friends and relatives		61 (50)	33 (23–61)†
Tourism		17 (14)	21 (13–34)
Tourists from endemic zone		14 (11)	NA
Business in endemic zone		11 (9)	25 (11–81)
Expatriate		9 (7)	832 (377–1189)
Military		4 (3)	18 (5–56)
Humanitarian		3 (2)	95 (22–NA)
Cryptic malaria		1 (1)	NA
Unknown		3 (2)	18 (16–NA)
Length of illness before starting artesunate; median d	123		4 (2–5)
Clinical data			
Temperature >40°C; mean temperature	118	9 (8)	39 (39–40)

Characteristic; value for last column	Total no. patients	No. (%) patients	Median (Q1–Q3)
Pulse rate >115 beats/min; mean cycles/min	102	30 (29)	104 (90–118)
Weight >130 kg; mean weight/kg	121	1 (1)	72 (61–81)
Severe malaria criteria at day 0			
Neurologic impairment			
Any signs	123	54 (44)	
Lethargy	123	46 (37)	
Confusion	123	39 (32)	
Multiple convulsion, >2 episodes in 24 h	123	1 (1)	
Glasgow Coma Scale <11; median score	113	16 (14)	15 (14–15)
Respiratory distress			
Any signs	123	14 (11)	
Respiratory rate >32 cycles/min; median cycles/min	63	9 (14)	20 (18–28)
Crackles	85	9 (11)	
Pulmonary infiltrates on chest x-ray	112	1 (1)	
PaO_2, mm Hg			90 (83–107)‡
PcO_2 mm Hg			34 (31–37)‡
PaO_2 <60 mm Hg; FiO_2 >21% with mechanical ventilation	115	13 (11)	8 (10)§
SpO_2 <91%; SpO_2 %	98	3 (3)	97 (96–99)¶
Cardiocirculatory impairment			
Any signs	123	25 (20)	
Shock (Spb ≤80 mm Hg);	102	9 (9)	
Peripheral signs of hypotension (with Sbp >80 mm Hg);	114	3 (3)	
Median Sbp, mm Hg			110 (92–125)
Median Dbp, mm Hg			64 (53–72)
Vasopressor supportive drug	112	10 (9)	
Spontaneous bleeding	123	1 (1)	
Purpuric lesions	113	2 (2)	
Macroscopic hemoglobinuria	123	17 (14)	
Jaundice and/or bilirubin >50 µmol/L	122	66 (54)	
Liver balance sheet			
Total bilirubin; median µmol/L	105		50 (26–81)
Alanine amino transferase; median UI/L	101		47 (29–83
Aspartate amino transferase; median UI/L	99		66 (37–103
Gamma-glutamyl transferase; median UI/L	94		65 (39–111)
Alkaline phosphatase; median UI/L	91		80 (63–121)
Splenomegaly	110	14 (13)	
Splenectomy	113	0	
Anemia (hemoglobin <7 g/dL or hematocrit <20%)	123	4 (3)	
Hemoglobin; median g/dL	116		12 (11–14)
Hematocrit; median %	88		36 (30–41)
Platelet count $\times 10^9$ cells/L; median count	114		42 (25–66)
Leukocyte count $\times 10^9$ cells/L; median count	109		6 (4–8)
Hypoglycemia (glucose <2.2 mmol/L); median mmol glucose/L	123	7 (6)	6 (5–7)#
Acidosis	120	14 (12)	
Plasma bicarbonate <15 mmol/L; median mmol bicarbonate/L	91	9 (10)	22 (19–24)
Acidemia pH <7.35; median pH level	69	10 (15)	7.4 (7.4–7.5)
Renal impairment	123		
Creatinine >120 µmol/L; median µmol serum creatinine/L	122	35 (29)	99 (75–143)†
Creatinine >265 µmol/L and/or blood urea >17 mmol/L; median mmol blood urea/L	123	16 (13)	7 (5–12)**
Hyperlactatemia (arterial lactate >1.8 mmol/L); median mmol plasma lactate/L	120	58 (48)	2 (2–4)††
Hyperparasitemia; median % infected erythrocytes	122		7 (2–11)
>4%		77 (63)	
>10%		33 (27)	
Hospital ward in which artesunate was prescribed and administered	123		
Intensive care unit		67 (55)	
Infectious disease/internal medicine unit		24 (20)	
Emergency unit		22 (18)	
Unknown		10 (8)	
First-line treatment for current severe malaria episode	123		
Artesunate		72 (59)	
Other drug		51 (41)	
Quinine		37	
Atovaquone + proguanil		6	
Artemether + lumefantrine		3	
Mefloquine		4	
Ceftriaxone		1	

Characteristic; value for last column	Total no. patients	No. (%) patients	Median (Q1–Q3)
Treatment by artesunate			
Median length of treatment, d	112		2 (2–3)
Median no. of doses per patient	109		5 (4–5)
Median total doses, mg	109		824 (600–990)
Died	123		6 (5)
Length of stay, median d			
Intensive care unit	114		1 (0–4)
Hospital	103		6 (4–8)
Other malarial drug treatment used after artesunate	123		
Yes		89 (72)	
Artemether + lumefantrine		58	
Atovaquone + proguanil		27	
Dehydroartemisinin + piperaquine		3	
Quinine, intravenous		1	
No		18 (15)	
Unknown		16 (13)	

*Dbp, diastolic blood pressure; NA, not available; Q1–Q3, quartiles 1–3; Sbp, systolic blood pressure.
†N = 106.
‡N = 61.
§N = 84.
¶N = 98.
#N = 85.
**N = 101.
††N = 83.

Technical Appendix Table 2. Complementary qualitative features of anemia, by non-PADH and PADH patterns, during follow-up for 72 patients with severe malaria treated with artesunate, France, 2011-2013

Pattern, feature	No. patients with feature/no. tested
Non-PADH pattern	
Positive DAT results	2/5†
Positive DAT results plus HIV positive	1
G6PD deficient	1/3§
Abnormal hemoglobin electrophoresis	3/6¶
Persisting renal insufficiency	5
Renal insufficiency plus HIV positive	1
HIV positive	3
Iron deficient	1/5
PADH pattern	
Positive DAT results	3/5‡
Positive DAT results plus HIV positive	1
G6PD deficient	0/5
Abnormal hemoglobin electrophoresis	0/6
Persisting renal insufficiency	1
Renal insufficiency plus HIV positive	0
HIV positive	1
Iron deficient	1/1

*DAT, direct antiglobulin test; G6PD, glucose 6 phosphate dehydrogenase; PADH, postartesunate delayed-onset hemolysis. PADH pattern was defined by 1) a new drop in the hemoglobin level after day 8 of treatment initiation and the appearance or reappearance of hemolytic markers (>10% drop in hemoglobin or >10% rise in lactate dehydrogenase levels) occurring any time between day 8 and the end of follow-up and/or 2) by any information in the medical chart referring to acute hemolysis occurring after day 8. The non-PADH pattern was defined by a hemoglobin nadir and a hemolysis peak occurring before day 8, with or without positive markers of hemolysis after day 8 and without a nadir or sudden drop of hemoglobin after day 8 as defined for the PADH pattern.
†Specification missing for 1 patient, and 1 patient was IgG positive by DAT.
‡Two patients IgM positive by DAT including 1 patient treated for a time with corticosteroid, and specification was missing for 1 patient.
§2 U/g hemoglobin.
¶ One case each of hemoglobin AS , hemoglobin AC, and heterozygous alpha-thalassemia.

Members of the French Artesunate Working Group

Mahamat Aba, Frederic Adnet, Jérôme Allyn, Muriel Alvarez, Dina Andriamanantena, Nadia Anguel, Claire Ara-Somohano, Nicolas Argy, Pierre Asfar, Camille Aubry, Elie Azoulay, Sylvain Jean-Baptiste, Philippe Barbe, Tonin Becret, Sorya Belaz, Bruno Bernot, Antoine Berry, Sylvestre Biligui, Frédéric Blanc, Martine Bloch, Jérome Bokobza, Emmanuelle Bosdure, Vlad Botoc, Lila Bouadma, Anissa Bouaziz, Olivier Bouchaud, Camille Bouisse, Pierre Bourgoin, Guillaume Breton, Marie Bruyere, Fabienne Caby, Monique Cambon, Fabrice Camou, Dorothée Carpentier, Serge Carreira, Enrique Casalino, Marion Caseris, Samuel Castro, Estelle Cateau, Amélie Chabrol, Nathalie Chartrel, Sarah Chemam, Frank Chemouni, Stéphanie Chevalier, Sylviane Chevrier, Christian Chidiac, Christophe Choquet, Liliane Cicéron, Delphine Claessens, Jerome Clain, Yves Cohen, Sandrine Cojean, Claire Contargyris, Rémi Coudroy, Naima Dahane, Pierre-Eric Danin, Franxois-Xavier Danlos, Sandrine Dautheville, Marc de Chambrun, Ludovic DeGentile, Pascal Delaunay, Pierre Dellamonica, Pierre Delobel, Alexandre Demoule, Ouda Derradji, Guillaume Pelée de St Maurice, Nathalie Desuremain, Edouard Devaud, Michel Develoux, Anna Lisa di Bari, Felix Djossou, Nathalie Dournon, Nicolas Dufour, Matthieu Dupont, Matthieu Duprey, Rémy Durand, Christelle Pomares Estran, Manuel Etienne, Loic Favennec, Albert Faye, Jean François Faucher, Bernard Faugère, Hélène Ferrand, Cécile Ficko, Clara Flateau, Arnaud Foucrier, Yonathan Freund, Sébastien Gallien, Gilles Gargala, Cécile Garnaud, Isabelle Gueit, Samia Hamane, Lilia Hamza, Pierre Hausfater, Benoit Henry, Isabelle Hermes, Sandrine Hours, Sidonie Hubert, Hervé Hyvernat, Houria Ichou, Véronique Joly, Gaelle Juillien, Philippe Karoubi, Judith Karsenti, Philippe Kraemer, Evgenia Krastinova, Sabah Kubab, Laurent Laine, Mathieu Laouisset, Henri Laurichesse, Sylvain Lavoué, Claire Lecompte, Marine Lecorre, Patrick Le guen, Guillaume Leloup, Virginie Lemiale, Gwénaël le Moal, Monique Lemoine, Nathalie Lerolle, Nicolas Lerolle, Yves Le Tulzo, Nicolas Libert, Coralie l'Ollivier, Jonathan London, Charles-Edouard Luyt, Chrystel Madre, Denis Malvy, Olivier Mangin, M. Marchand, Bruno Marchou, Vincent Mardelle, Eric Mariotte, Pierre Marty, Sophie Matheron, Sarah Mattioni, Dominique Maubon, Adeline Max, Virginie Maxime, Matthieu Mechain, Guillaume Mellon, Stéphane Mérat, Audrey Merens, Laurence Million, Matthieu Million, Philippe Minodier, Camille Moisant, Jean-Michel Molina, Viviane Montout, Satar Mortaza, Emilie Mosnier, Nacira Mouffok, Oussama Mouri, Grégoire Muller, Michel Muller, Jean Benjamin Murat, Jérome Naudin, David Osman, Philippe Parola, Antoine Parrot , Olivier Paut, Julie Pernet, Gilles Perrin, Christian Perrone, Eric Peytel, Luu-Ly Pham, Renaud Piarroux, Eric Pichard, Stéphane Picot, Marc Pihet, Jean-Etienne Pilo, Denis Pons, Hélène Prodanovic, Céline Pulcini, Lauren Pull, Didier Raffenot, Loic Raffray, Pascal Ralaimazava, Bruno Ralec, Patrick Ray, Marie Catherine Receveur, Matthieu Revest, Bruno Riou, Christophe Rioux, Olivier Rogeaux, Philippe Sachs, Carole Sarda, Claudine Sarfati, Véronique Sarrasin, Hélène Savini, Fabrice Simon, Jean-Yves Siriez, Bertrand Souweine, Alina Stoian, Fabien Taieb, Mercedes Tatay, Pierre Tattevin, Bertrand Texier, Michel Thibault, Jean-Marc Thouret, Françoise Benoit Vical, Nicolas Vignier, Daniel Vittecoq, Michel Wolff, Francis Xeridat, Yazdan Yazdanpanah, and Julie Zundel.
